# Supplementary material for: ImmuCellAI: A Unique Method for Comprehensive T‐Cell Subsets Abundance Prediction and its Application in Cancer Immunotherapy
Source: Adv Sci (Weinh). 2020 Feb 11;7(7):1902880. doi: 10.1002/advs.201902880 (PMC7141005; doi:10.1002/advs.201902880)
Supplement: Supplementary file 1 — Supporting Information [file ADVS-7-1902880-s001.pdf]

Copyright WILEY-VCH Verlag GmbH & Co. KGaA, 69469 Weinheim, Germany, 2020.

ADVANCED SCIENCE
Open Access

Supporting Information

for *Adv. Sci.,* DOI: 10.1002/advs.201902880

ImmuCellAI: A Unique Method for Comprehensive T-Cell Subsets Abundance Prediction and its Application in Cancer Immunotherapy

*Ya-Ru Miao, Qiong Zhang, Qian Lei, Mei Luo, Gui-Yan Xie, Hongxiang Wang, and An-Yuan Guo**

Supporting Information

Figure S1 Direct comparison (Pearson correlation analysis) between cell fractions predicted by ImmuCellAI and the real cell abundance listed in Fig. 2b on PBMCs of samples from healthy individuals (a) and patients with AML (b).

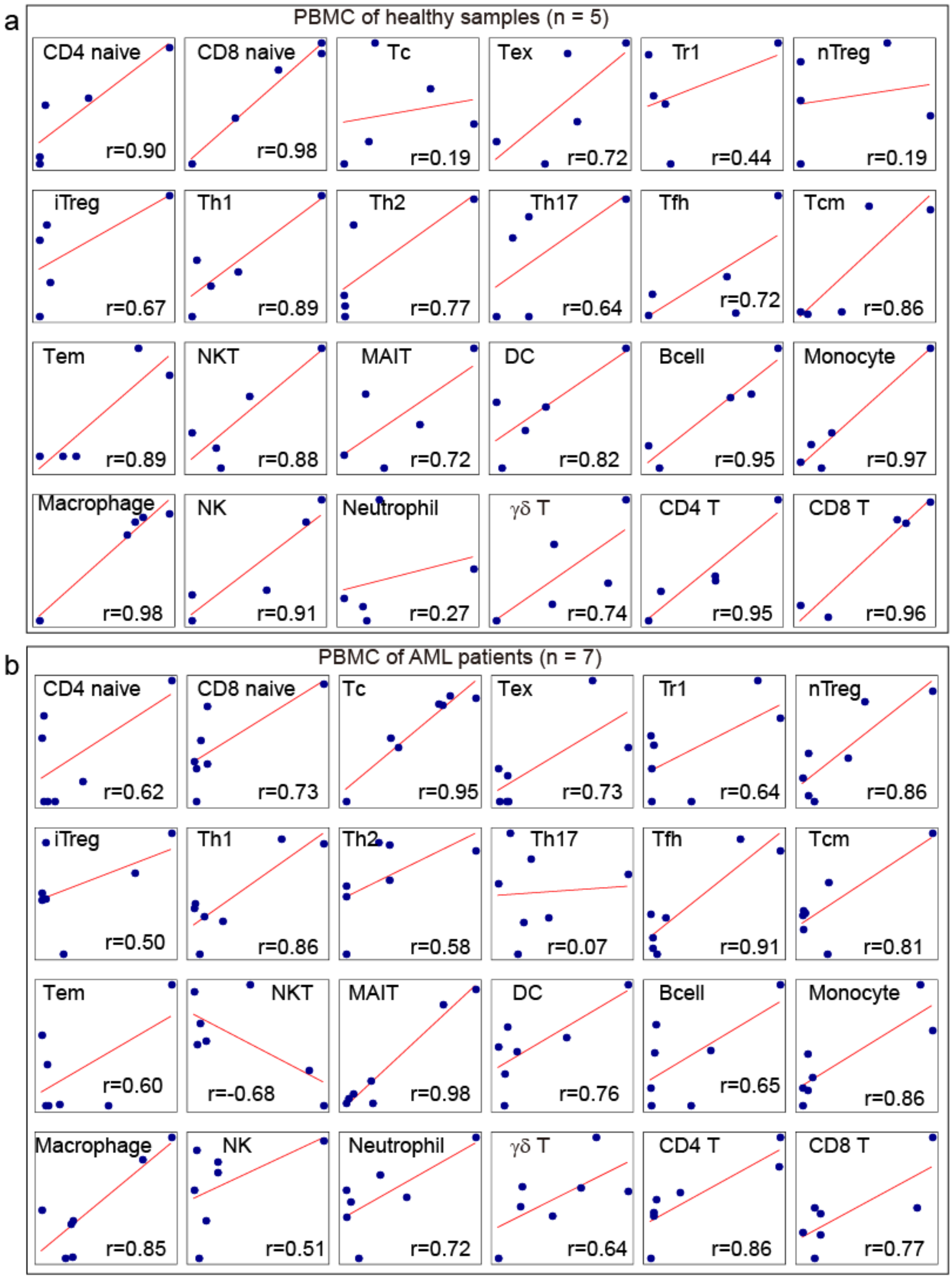

Figure S2 Direct comparison between cell fractions predicted by ImmuCellAI and the real cell abundance listed in Fig. 2c and 2d in public datasets estimated by RNA-Seq (a) and microarray (b).

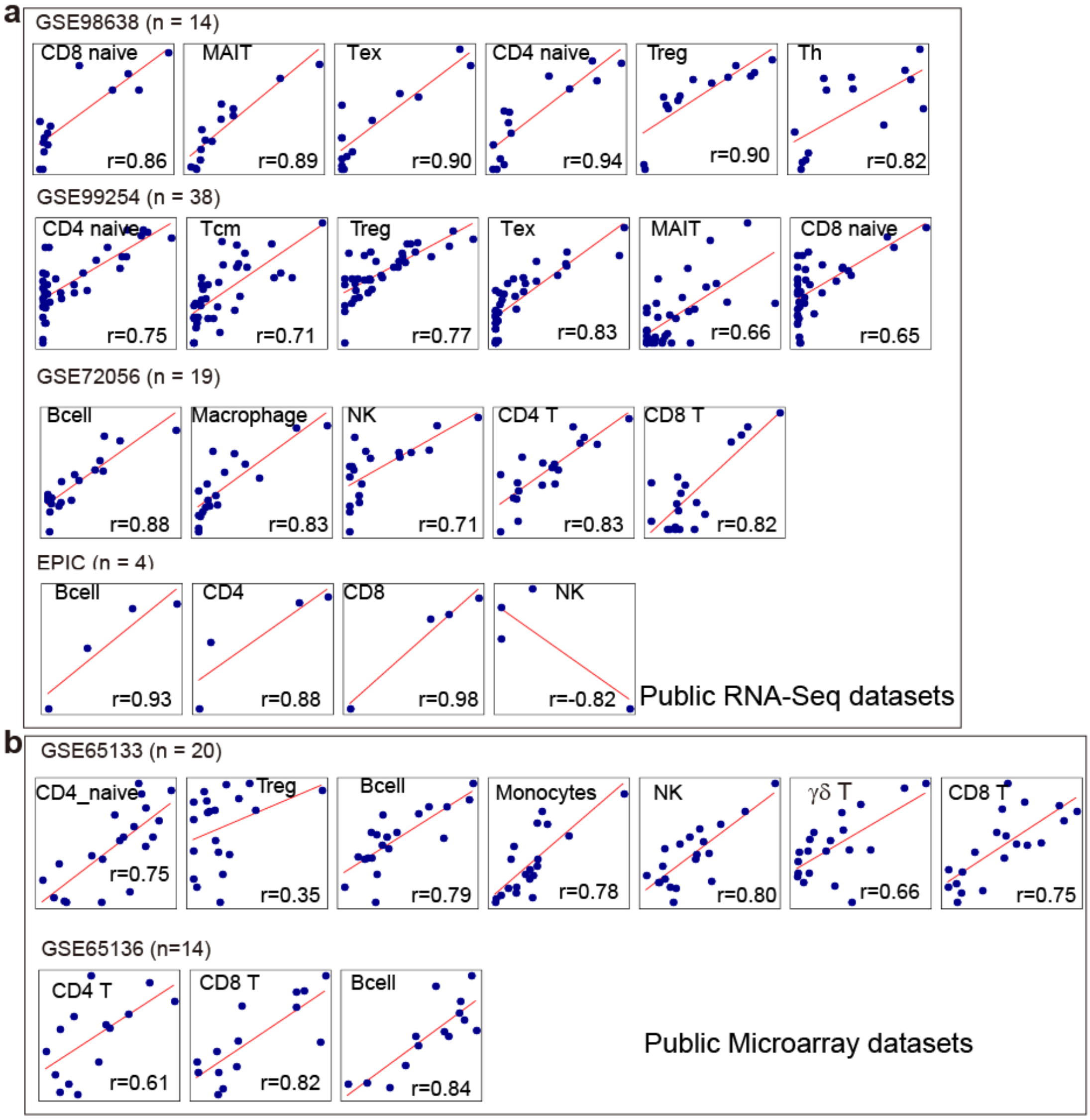

Figure S3 Partial correlation analysis of the infiltration of immune cells between the nidus and adjacent tissues, taking age and gender into consideration

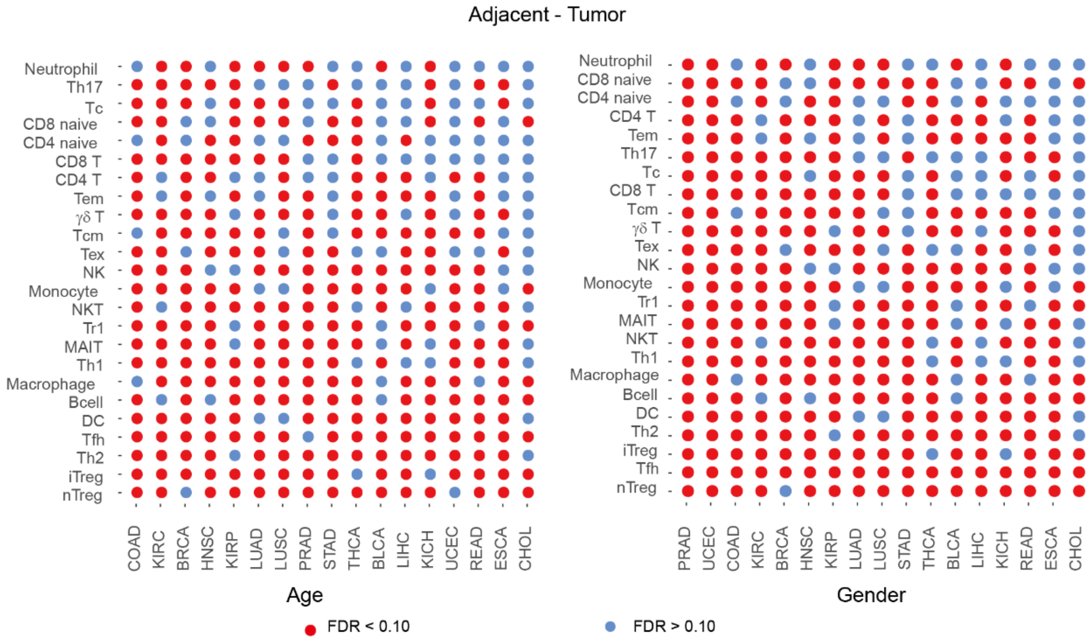

Figure S4 Kaplan–Meier curves of melanoma (SKCM) by the infiltration of 12 immune cell types including Tfh, γδ T, Tc, Neutrophil, Th1, NKT, CD8 T, Th2, CD8 naïve, NK, Th17, and iTreg.

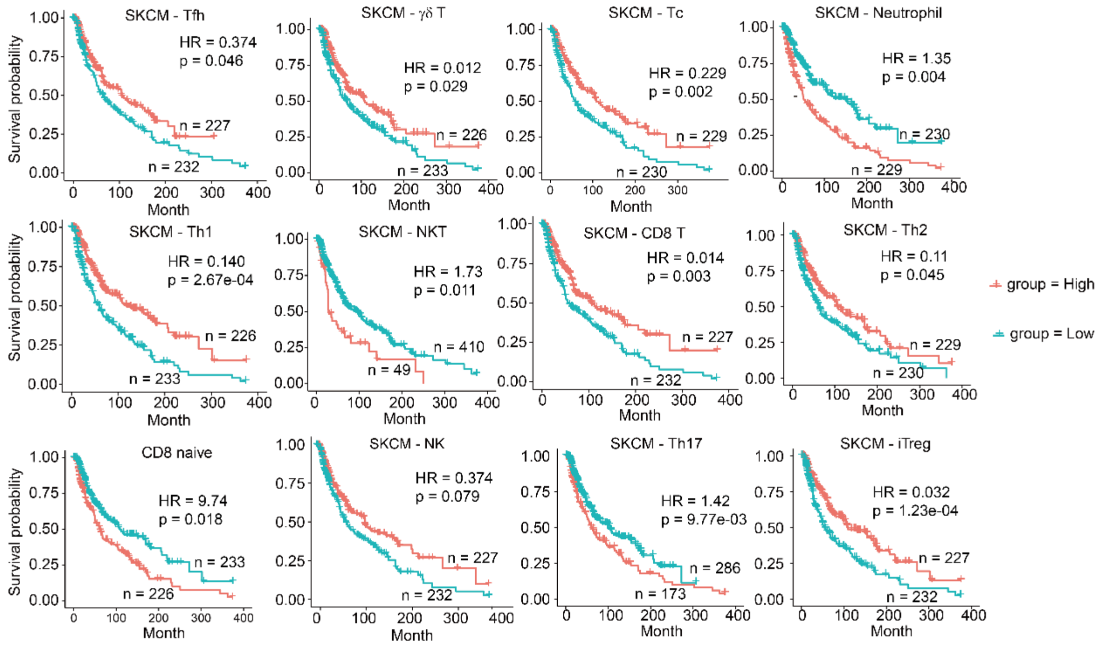

Figure S5 Partial correlation analysis of the infiltration of immune cells and clinical factors (age and gender)

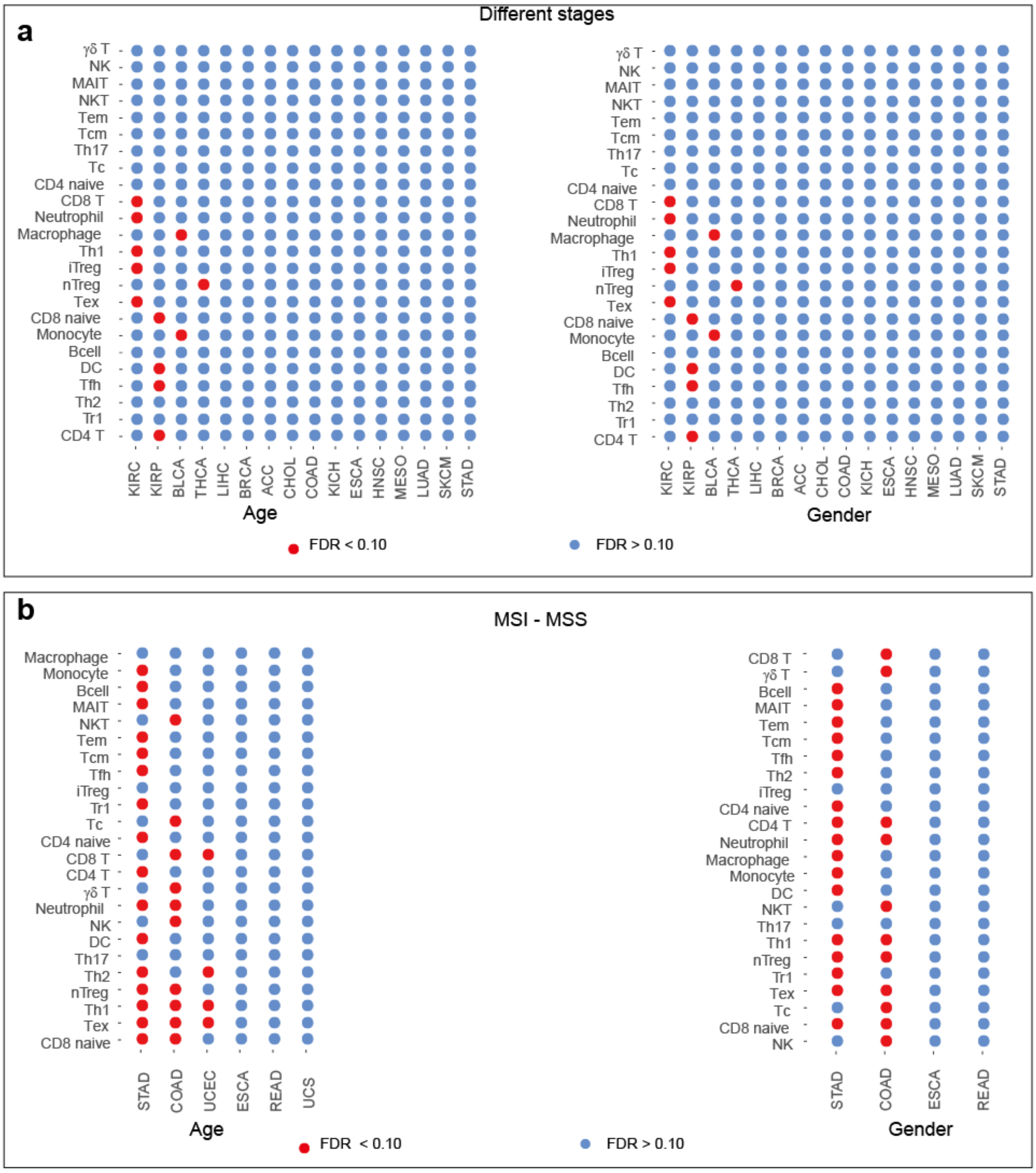

Figure S6 Association of the infiltration of antitumor (a) and tumor suppressor (b) immune cells with microsatellite instability (MSI) status in COAD and STAD.

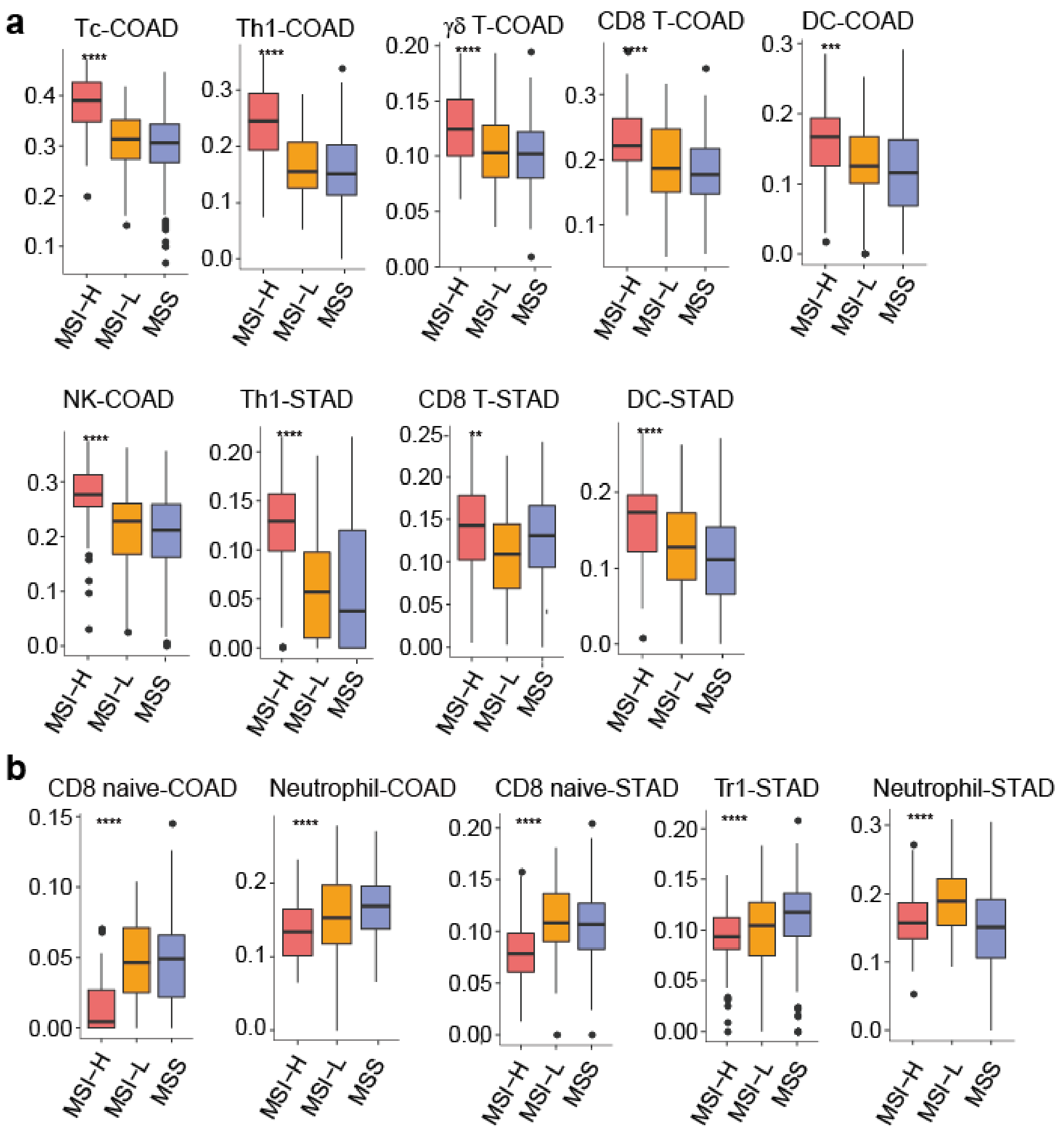

Figure S7 The abundance of tumor-infiltrating Tex, Th1, iTreg, and CD8$^+$ T cells elevated with the clinical stage in KIRC

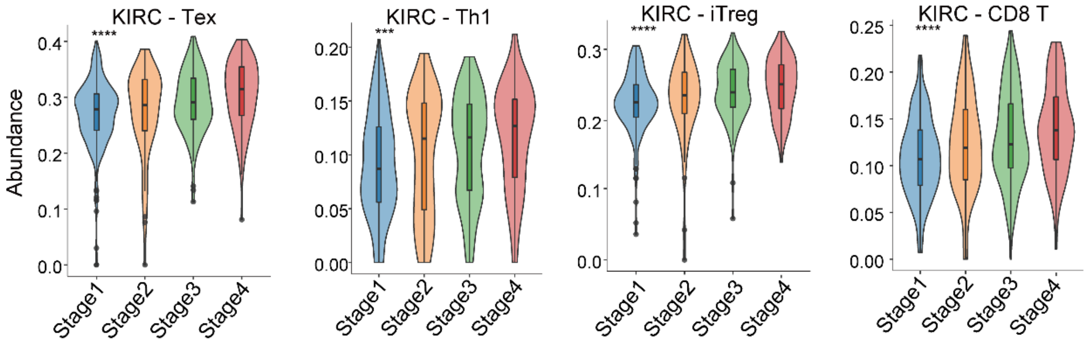

Table S1 All marker genes used in ImmuCellAI

Table S2 A list of immune cell reference expression profiles collected from the GEO database

Table S3 Characteristics of the patients and healthy donors

Sample ID	Gender	Age	Sample source	Trait
L1	Female	55	PBMC	AML
L2	Female	53	PBMC	AML
L3	Female	77	PBMC	AML
L4	Male	47	PBMC	AML
L5	Female	59	PBMC	AML
L6	Male	68	PBMC	AML
L7	Male	70	PBMC	AML
A	Female	30	PBMC	Healthy
B	Male	25	PBMC	Healthy
C	Male	28	PBMC	Healthy
D	Male	27	PBMC	Healthy
E	Female	45	PBMC	Healthy

Table S4 Immune cell antibodies used in flow cytometry

**Cell type**	**Antibody**
Bcell	CD45+CD19+
DC	CD11c+HLA-DR+

Macrophage	CD45+CD11b+
Monocyte	CD45+CD14+
Neutrophil	CD66b+CD11c+
NK	CD45+CD56+CD3-
NKT	CD45+CD3+CD56+
CD4 naïve	CD3+CD4+CD45RA+CCR7+
CD4 T	CD3+CD4+
iTreg	CD4+CD25+FOXP3+Helios-
nTreg	CD4+CD25+FOXP3+ Helios+
Tfh	CD4+CD25-CXCR5+PD-1+
Th1	CD45+CD4+IFN-r
Th17	CD45+CD4+IL-17a
Th2	CD45+CD4+IL-4
Tr1	CD4+CD25-LAG3+
CD8 naïve	CD3+ CD8+CD45RA+ CCR7+
CD8 T	CD3+CD8+
Exhausted	CD45+CD3+CD8+PD-1+
Cytotxic	CD3+CD8+CD57+TCRVβ+
Central memory	CD45+CD3+CD45RO+CCR7+
Effector memory	CD45+CD3+CD45RO+CCR7-
Gamma delta T（γδT）	CD3+TCRVδ2+

MAIT	CD3+ CD161++ Va7.2 TCR+

Table S5 Antibody and its corresponding clone id used in flow cytometry

**Antibody**	**Clone id**
anti-CD45-APC-H7	clone 2D1
anti-CD4-BB515	clone RPA-T4
anti-CD8-PE	clone RPA-T8
anti-CD3-PerCPCy5.5	clone SP34-2
anti-CD25-PECy7	clone M-A251
anti-CD279-APC	clone MIH4
anti-CCR7-Alexa Fluor 647	clone 3D12
anti-IL-4-PE	clone 8D4-8
anti-IFN- γ - PercpCy5.5	clone 4S.B3
anti-IL-17-Alexa Fluor 647	clone SCPL1362
anti-Helios-Alexa Fluor 647	clone 22F6
anti-FoxP3-BB700	clone 236A/E7
anti-LAG-3-PE	clone T47-530
anti-CXCR5-BB700	clone RF8B2
anti-CD56-PECy7	clone B159

anti-CD19-APC	clone HIB19
anti-HLA-DR-PECy7	clone G46-6
anti-CD11c-PE	clone B-ly6
anti-CD66b-PerCPCy5.5	clone G10F5
anti-CD11b-APC	clone M1/70
anti-CD14-FITC	clone M5E2
anti-CD45RA- PECy7	clone HI100
anti-CD45RO- PECy7	clone UCHL1
anti-CD57-FITC	clone NK-1
anti-TCR-V β 2-PE	clone REA654
anti-V δ 2 TCR-PE	clone B6
anti-CD161-FITC	clone DX12
anti- TCR-Va7.2-APC	clone REA179

Table S6 Proportion of immune cells measured by flow cytometry for the PBMC of AML and healthy donors

Table S7 The real immune cell fraction calculated from single-cell barcode of GSE98638

**sample**	**CD8 naive**	**MAIT**	**Exhausted**	**CD4 naive**	**Treg**	**Th**
p0205p	0.121	0.071	0.000	0.558	0.080	0.027
p0205t	0.009	0.037	0.092	0.005	0.436	0.179

p0322n	0.005	0.405	0.000	0.074	0.005	0.279
p0322p	0.088	0.137	0.003	0.245	0.232	0.046
p0322t	0.010	0.138	0.121	0.036	0.363	0.183
p0407n	0.013	0.100	0.017	0.071	0.088	0.363
p0407p	0.107	0.036	0.008	0.433	0.119	0.024
p0407t	0.000	0.040	0.048	0.082	0.072	0.401
p0508n	0.005	0.283	0.000	0.052	0.009	0.344
p0508p	0.156	0.051	0.000	0.452	0.126	0.037
p0508t	0.004	0.006	0.203	0.052	0.376	0.102
p1116n	0.000	0.100	0.000	0.021	0.170	0.390
p1116p	0.048	0.024	0.000	0.335	0.287	0.004
p1116t	0.003	0.020	0.187	0.003	0.313	0.093

Table S8 The real immune cell fraction calculated from single-cell barcode of GSE99254

Sample	CD4 naive	Tcm	Treg	Exhausted	MAIT	CD8 naive
P0616A_P	0.158	0.055	0.060	0.000	0.004	0.070
P0616A_T	0.004	0.015	0.126	0.004	0.024	0.007
P0616P_P	0.154	0.101	0.000	0.000	0.016	0.202
P0616P_T	0.000	0.000	0.017	0.111	0.000	0.017
P0617_N	0.003	0.091	0.023	0.000	0.000	0.003
P0617_P	0.063	0.194	0.063	0.004	0.004	0.026

P0617_T	0.000	0.015	0.031	0.037	0.031	0.002
P0619_N	0.011	0.018	0.060	0.021	0.000	0.000
P0619_P	0.101	0.105	0.072	0.000	0.018	0.080
P0619_T	0.003	0.003	0.145	0.222	0.003	0.000
P0706_P	0.010	0.058	0.010	0.000	0.000	0.019
P0706_T	0.028	0.051	0.252	0.126	0.008	0.002
P0729_N	0.003	0.000	0.016	0.006	0.003	0.013
P0729_P	0.020	0.081	0.054	0.000	0.000	0.071
P0729_T	0.000	0.004	0.033	0.050	0.017	0.012
P0913_N	0.049	0.038	0.000	0.011	0.000	0.011
P0913_P	0.084	0.158	0.134	0.000	0.000	0.039
P0913_T	0.029	0.006	0.243	0.049	0.006	0.004
P1010_N	0.005	0.028	0.133	0.000	0.000	0.005
P1010_P	0.049	0.171	0.046	0.000	0.000	0.003
P1010_T	0.000	0.008	0.299	0.053	0.002	0.000
P1011_N	0.000	0.000	0.000	0.231	0.000	0.000
P1011_P	0.202	0.154	0.123	0.003	0.003	0.084
P1011_T	0.000	0.004	0.169	0.127	0.002	0.000
P1118_N	0.005	0.047	0.026	0.005	0.000	0.000
P1118_P	0.133	0.254	0.023	0.000	0.000	0.152
P1118_T	0.007	0.028	0.113	0.035	0.007	0.000
P1120_N	0.000	0.000	0.000	0.073	0.049	0.000

P1120_P	0.124	0.084	0.084	0.000	0.026	0.061
P1120_T	0.001	0.006	0.201	0.128	0.011	0.001
P1202_N	0.004	0.015	0.045	0.015	0.023	0.004
P1202_P	0.121	0.088	0.058	0.000	0.006	0.000
P1202_T	0.007	0.024	0.164	0.073	0.004	0.011
P1208_P	0.164	0.085	0.141	0.000	0.009	0.141
P1208_T	0.005	0.014	0.153	0.032	0.014	0.000
P1219_N	0.070	0.000	0.000	0.000	0.009	0.000
P1219_P	0.131	0.049	0.087	0.000	0.038	0.038
P1219_T	0.000	0.000	0.055	0.008	0.016	0.000

Table S9 The real immune cell fraction calculated from single-cell barcode of GSE72056

Sample	Bcell	Macrophage	Endothelial	NK	CD4 T	CD8 T
Patient53	0.050	0.066	0.066	0.061	0.189	0.112
Patient58	0.024	0.012	0.000	0.024	0.116	0.387
Patient59	0.018	0.009	0.000	0.000	0.000	0.000
Patient60	0.372	0.019	0.000	0.041	0.130	0.126
Patient65	0.052	0.021	0.000	0.000	0.096	0.344
Patient67	0.159	0.000	0.008	0.008	0.218	0.194
Patient71	0.023	0.015	0.000	0.000	0.036	0.145
Patient72	0.168	0.000	0.005	0.005	0.291	0.103

Patient74	0.079	0.028	0.000	0.006	0.041	0.415
Patient75	0.013	0.010	0.003	0.000	0.029	0.554
Patient78	0.013	0.000	0.000	0.000	0.000	0.000
Patient79	0.088	0.000	0.003	0.001	0.038	0.186
Patient80	0.101	0.002	0.057	0.010	0.179	0.103
Patient81	0.013	0.004	0.009	0.004	0.039	0.109
Patient82	0.019	0.039	0.010	0.029	0.122	0.069
Patient84	0.145	0.140	0.005	0.038	0.123	0.117
Patient88	0.044	0.106	0.003	0.023	0.094	0.122
Patient89	0.215	0.051	0.006	0.002	0.049	0.230
Patient94	0.171	0.007	0.057	0.002	0.164	0.078
